# Mr. Thomas, in Reply to Mr. Dawson

**Published:** 1807-06

**Authors:** Charles Thomas

**Affiliations:** H. M. Ship, Resolue


### To the Editors of the Medical and Phyſical Journal.

GENTLEMEN,

### To the Editors of the Medical and Phyſical Journal.

GENTLEMEN,

IN noticing the contents of Mr. Dawson's last paper, I shall make it a principal aim to take up as little time as the nature of the subject will allow. I also assure you, that relating to this subject I shall not have to solicit another place in your Journal. To this determination I am led by an entire conviction that its pages, destined to purposes so much more valuable, would be encumbered and vi-

 tiated tiated by useless, protracted disputation. This remark applies to both parties; therefore Mr. Dawson must not be offended, because it comprehends the fruit of his own ingenious mind, which perhhaps, in a pleasing mood, he often anticipates; as well as the crudities of one, like myself, so far removed from the character described in a clause of his quotation from the facetious part of Cornwall. The points at issue between us, do not involve the general management of stricture. The concessions, which Mr. Dawson remarks, I have so liberally made to him, must imply some coincidence of opinion, or else go for nothing. All I considered objectionable in Mr. Dawson's first communication, was the unqualified censure bestowed on the early part of the treatment, pursued by Dr. Bellamy in the case of Mr. Sharp, and the "supposition" of a ruptured urethra by the introduction of the catheter. As the last altogether, and the former in part, are abstract circumstances, merely relating to this single case, they cannot be supposed as likely to conduct us to any further useful investigation; on which account I retire from future controversy, with a mind confirmed in the validity of my former statement, but open to all possible conviction, should Mr. Dawson be able to produce something a little more cogent than appears in his late reply.

Mr. Dawson may impute my adherence to the opinion I originally gave about those indications of inflammation, which he now more strenuously contends for than ever, to unreasonable doubt, or inveterate prejudice. But, from whatever cause it may proceed, I do assure him, all his efforts have been completely ineffectual in correcting my imputed errors. He merely recapitulates the same supposed signs of inflammation, when Mr. Sharp first complained, which he gave us before, with the addition, it is true, of a very notable one, consisting of nothing more or less, than "an affection of his general health!" But, on a chronic tumour, produced by stricture in the urethra, slight mechanical pressure may occasion "pain;" in an urethra, almost impervious, "internal distress, a sense of fulness and aching," may be felt when the parts are at rest, and still more when urine or fæces are evacuated; and in a subject, like Mr. Sharp, ten thousand sympathies of the constitution may produce "an affection of the general health," without having recourse to Mr. Dawson's favourite notion of prevailing inflammation. Indeed, if any weight can be attached to this supplementary circumstance of "an affection of the general health," it can hardly be collected collected from Dr. Bellamy's account of Mr. Sharp on the first day. Dr. Bellamy simply states, that he "looks ill," but makes no mention of constitutional ailment, as observable from the pulse, or any other of the sources, usually indicating it. Mr. Dawson, in endeavouring to establish the premises on which part of his reasoning is to be founded, seems to invite our attention emphatically to this new discovery. But it must be obvious to the most cursory observer, that this intended key-stone will not fit, so as to afford the superstructure any support. After this enumeration of Mr. Sharp's symptoms, we are furnished with Mr. Dawson's own selection of those which mark inflammation. Among them, "great irritability" is particularly noticed. Now, I beg to ask Mr. Dawson, whether we may not rationally conclude, that a man so long subject to a most distressing and painful disease, as Mr. Sharp unfortunately was, might not be the "very victim of irritability," without ever experiencing any recurrence of inflammation after that attack of it, which was most probably the original cause of his stricture.

Mr. Dawson calls the attention of his readers to the circumstance of a fluctuation of matter being evident in the tumour on the 25th of January, 1806, the fourth day after Mr. Sharp came under Dr. Bellamy's care; and seems to offer it as an additional evidence of inflammation on the 22d. But surely, without wandering very far away in the paths of conjecture, we may fairly attribute the formation of matter so early as two days after the first appearance of inflammation to the extreme susceptibility, which the parts have to take on an inflammatory action, when affected with stricture. As Mr. Dawson truly states, the inflammation rapidly increased: this it did from the 24th; but never could at any former period, because I am still convinced it never before existed. Here, therefore, the impression before made on my mind recurs with increased force, in favour of the opinion, that the "termination of the case did not prove the swelling to be no chronic enlargement." External induration, and some degree of swelling, are almost invariable concomitants of stricture. In the case of Mr. Sharp, it was evidently so. Six months before, these appearances were treated with advantage to the patient by the same plan, which has been so much condemned.

Mr. Dawson has entered pretty largely and correctly into the consideration of the superior advantages, derivable from mercury, when administered through the medium of the the system, than by the application of it to a diseased part. As a general principle this may be admitted without reserve; but I am sure the successful experience of very many practitioners will not allow them to sanction Mr. Dawson's idea universally. Yet why introduce this comparative statement about the two modes of administering mercury? I hope it was for a better purpose than a mere display of the author's physiological acumen. It certainly is but little, if at all, connected with a discussion of the present case; I should naturally infer from the whole tenor of Mr. Dawson's observations, that this powerful agent is prescribed by him in every shape. The unhappy manner in which this digression is introduced, would lead a more captious opponent than myself to aver, that Mr. Dawson had said, Dr. Bellamy should have made his patient rub in. However, I altogether disavow such an insinuation, and sincerely believe Mr. Dawson clear of so much inconsistency. He only appears here to have lost his usual correct controul over his pen. Mr. Dawson prsses me very closely with his interrogatories on the subject of following, under the same circumstances, the treatment which was adopted the two first days in Mr. Sharp's case. They are urged with such apparent confidence, as to bespeak a conviction in their author, that I must renounce my former opinion. The answer I shall give to one of these will be no renunciation of it. "Would he employ warm fomentations?" says Mr. Dawson. "No," is my reply. "Would he use more than five grains?" "Yes, certainly." In relation to the last of these, Dr. Bellamy's practice may be considered as rather inert, but by no means mischievous. Though if he regulated the quantity of ointment by his former success, we have no right to think his practice at all defective. As to the fomentations, I have always regarded them as forming so unimportant a part of the treatment, to be employed or not with so little probable benefit or detriment to the patient, that I never should have used them; though it would be injustice to Dr. Bellamy not to remark, that there is some force in his reasoning in favour of their adoption; that "fomenting a painful swelling, with thick covering, will procure ease, and greatly assist the introduction of ungt. hydrarg." This is analogous to the practice of conjoining the warm bath with the use of mercury.

Mr. Dawson says, I am mistaken in asserting, that he "labours" to fix the charge on Dr. Bellamy, of having forced his catheter through Mr. Sharp's urethra. It certainly tainly must be so, since Mr. Dawson solemnly denies it. But, if by that term be meant an exertion of the faculties to perform any act of difficult execution, I cannot help thinking, that when I wrote, I had good ground to conclude it was not misapplied. Mr. Dawson would have his readers suppose he has been forced, by what I have advanced, to sit down calmly, to convict (as he calls it) a gentleman of having committed an injury which is reprehensible. But I would direct their attention to the extract he has made from his own remarks. Let it be read in its original place; viewed in the connection it there occupies; and weighed in the balance of a just and liberal interpretation. Then let it be declared, whether in any subsequent observations Mr. Dawson could more strongly confess his belief in the existence of such an injury than in the passage alluded to, which is now denominated a mere "supposition." No possible form of words can more strongly express a writer's conviction of the truth or reality of the subject, about which he inquires, than an interrogation, negatively constructed, like the following: "Was not an opening made through the urethra into the abscess by the point of the catheter!"

However inapplicable the term might be when I used it to describe Mr. Dawson's "supposition;" yet, I think, he must allow its peculiar propriety in reference to the attempt he makes to establish the charge in his reply; and I have no doubt, however contrary to Mr. Dawson's own hopes, it will be generally considered as labour in vain.

"Hoc opus, hic labor est."

Mr. Dawson has introduced no little confusion in his attempts here, by aiming at a great deal more than he can accomplish. He has, in fact, declared, that the circumstance of Dr. Bellamy's having occasioned a loss of continuity in Mr. Sharp's urethra, by the catheter, can be satisfactorily proved; whether such an accident be considered either as the "immediate" or "remote" consequence of violence. I have less to do, as by me it has been considered in the former point of view already. My assumption, I still think, is not open to very many exceptions. Mr. Dawson does not say, that the remote mischief is more frequent. There is some difference in the dates, mentioned in Mr. Dawson's annotations on my remarks concerning the improbability of the event he contends for, and those to be found in the remarks themselves, owing to an obscurity in the original narrative. The month of February bruary is also written by both instead of January. But this does not affect the validity of the conclusion I have drawn, since in the observations of each we find the space of "forty-eight hours," between the last introduction of the catheter and the discovery of urine passing through the opening in the perinæum. Mr. Dawson directs me to remember, that on the 30th, urine came from about the bulb of the urethra, the identical place where the point of the catheter encountered obstruction. Will Mr. Dawson also remember, that this is the identical place where obstruction and inflammation were always particularly seated? No urine could have come away before. Had it done so, the patient must have felt it; for though, as remarked by Mr. Dawson, it is said the urine, which passed, gave him no pain, yet, it is also said, he had a "sense of trickling and heat." This sense of heat would have been particularly observable when the poultices were become a little cold. The attendants must have observed a discharge of urine, if there had been any through a preternatural opening. Dr. Bellamy, whose humane attention would lead him often to the bed-side of his patient, must have observed it. Let me here repeat, that what is mentioned on the 30th, of "obstructions being felt breaking down under the catheter," refers to a time when the discharge of urine had been previously discovered, and the parts attained such a state of extensive disease, that we may reasonably suppose, as I before have done, that this sensation was produced by coagula in the urethra.

Let us now see how far Mr. Dawson has been successful in establishing his point, on the ground that "immediate inflammation and consequent sloughing" may be the effect of violent mechanical injury done to the urethra. He observes, that all the mischief was probably produced on the 28th. Inflammation was then excited, which in the course of about two days was followed by sloughing.—That inflammation led to all the subsequent mischief I fully admit; but then it was "spontaneous" inflammation, and not the effect of any external agency whatever. That it had existed for days before is allowed by all. I never disputed its presence from the 24th. Mr. Dawson urges, with much earnestness, the circumstance of blood coming away with the catheter on the 28th. But is it not probable, that in the diseased state of the urethra then, bleeding would be excited, were the catheter introduced in the most gentle way? Let us remark the description given of the abscess on the 26th; "nearly as large as a fist, and extending extending quite to the anus;" and that on the following day it broke. On the 29th, the bulb of the urethra was nearly exposed. The ravages made on the neighbouring parts were equally formidable. Would it not be a matter of surprise then for the urethra to escape? The case to me is quite clear. I have no doubt the morbid action took hold of this canal as well as the parts contiguous to it, till a sloughing of it happened on the 29th.

Thus the discharge of urine by the perinæum was the effect of inflammation, precisely in the same way that fistulæ are usually produced. I very much wonder Mr. Dawson's acuteness did not prevent him from attributing it to a different cause; and even if his suspicions were ever so well grounded, that a sense of delicacy had not restrained him from mentioning it altogether. Mr. Dawson asks, where is my boasted refutation? I answer, that it remains unmoved by all his efforts to overturn it; and that the day has not yet arrived for his "damning proof" to make its appearance.

The concluding topic of Mr. Dawson's reply I revert to with the utmost unwillingness. I much wished to have forborn saying a single word more on a subject not grateful to that gentleman's feelings; but the reference I made to a case, related in this Journal some time since, appears to have been received in a way so opposite to my intentions; and Mr. Dawson, with much apparent eagerness and anxiety, having required a further discussion of some points connected with it, I deem it due both to him and myself to take a little notice of them, though I do it most relunctantly.

From his paper on that case, as well as his first on Mr. Sharp's, I do consider it can be shown, that Mr. Dawson was in both instances premature in his decision. So soon after receiving an account of the Case, to offer even "conjectures," which were in direct opposition to the conclusions of three respectable practitioners, who by attendance on the patient had the evidence of their senses as well as their understandings to direct them, while the case to Mr. Dawson could only be a matter of speculative investigation, is sufficient to establish what I have asserted. The case was calculated to make the most experienced veteran in the obstetric art to pause, ponder, and seek new light. Yet we find the intuitive mind of Mr. Dawson penetrates obscurity, and gives a ready solution of every difficulty, within a few days after he became acquainted with the circumstances of it. Mr. Dawson will gain nothing thing by referring his readers to the letters which passed on that occasion; they may learn there, that the conjectures are "handsome," in respect to the style and manner of the author to the gentlemen concerned; that they are "ingenious," like every thing else from Mr. Dawson: but after the most diligent research, they will find nothing in contradiction to the idea of their premature promulgation. What has been here advanced may be applied with equal propriety to the case of Mr. Sharp; for to comment with severity on that case, at a time when it was but in part published, may be well termed a too hasty decision. It was the close affinity between these two cases, as to the manner in which they were reviewed by Mr. Dawson, that brought the former fresh to my recollection.

After dismissing this ungrateful part of my subject, will Mr. Dawson have the goodness to admit my solemn asseveration, when I declare, that if I could have conceived my allusion to the case of singular delivery would have produced the disquietude in his mind, which it seems to have done, that I never would have mentioned it at all? No advantage, derivable from it to the cause I have espoused, should have induced me to have made any reference to it.

In addition to my other concessions, I most readily allow Mr. Dawson the full benefit of another. The opinion he states of Dr. Bellamy's professional character has been quoted by me in an inaccurate way. I allude to the precise terms employed by Mr. Dawson; for I am not entirely convinced of being so far from the true, legitimate meaning, which may be inferred from their relation to each other, as he represents. And he must not imagine, because his perceptions and those of another happen to be different on this point, that both veracity and candour may not reign as extensively in the mind of that person as in his own.

An impartial retrospect of what has passed between us, will, I think, convince Mr. Dawson, that he has occupied untenable ground. His candour will lead him to this conclusion. He possesses, I have no doubt, that estimable quality, as well as many others. His respectable talents have afforded different contributions to the Medical and Physical Journal, which I have perused with pleasure; and I hope often to experience the same gratification. He has my respects; and I should be sorry if any thing has occurred to deprive me of a small share of his.

I am, &c.

CHARLES THOMAS.

H. M. Ship, Resolue, March 11, 1807.

